# Supplementary material for: Activation of a neural stem cell transcriptional program in parenchymal astrocytes
Source: eLife. 2020 Aug 3;9:e59733. doi: 10.7554/eLife.59733 (PMC7440914; doi:10.7554/eLife.59733)
Supplement: Transparent reporting form [file elife-59733-transrepform.docx]

***eLife’s* transparent reporting form**

We encourage authors to provide detailed information *within their submission* to facilitate the interpretation and replication of experiments. Authors can upload supporting documentation to indicate the use of appropriate reporting guidelines for health-related research (see [EQUATOR Network](http://www.equator-network.org/%20)), life science research (see the [BioSharing Information Resource](https://biosharing.org/)), or the [ARRIVE guidelines](http://www.plosbiology.org/article/info:doi/10.1371/journal.pbio.1000412) for reporting work involving animal research. Where applicable, authors should refer to any relevant reporting standards documents in this form.

If you have any questions, please consult our Journal Policies and/or contact us: [editorial@elifesciences.org](mailto:editorial@elifesciences.org).

**Sample-size estimation**

- You should state whether an appropriate sample size was computed when the study was being designed
- You should state the statistical method of sample size computation and any required assumptions
- If no explicit power analysis was used, you should describe how you decided what sample (replicate) size (number) to use

Please outline where this information can be found within the submission (e.g., sections or figure legends), or explain why this information doesn’t apply to your submission:

Before each experiment, sample sizes were estimated in the following ways:

Cx30-CreER scRNA-seq dataset:

We selected four experimental conditions for this dataset, choosing time points that span the duration of the neurogenic process. This is specified in the Methods section (“Library preparation and sequencing for the Cx30-CreER dataset”), and the reasoning is laid out in the Results section (section “Transcriptome-based reconstruction of neurogenesis by striatal astrocytes”).

AAV-Cre scRNA-seq dataset:

We selected a single time point at which to perform scRNA sequencing, a time point that encompasses both activated neurogenic astrocytes, transit-amplifying cells and neuroblasts (but not ground-state astrocytes). Because 10X Chromium generates thousands of single cell libraries, we pooled 11 mice for this dataset. These details are specified in the Methods section (“Sample preparation for the AAV-Cre dataset”) and the reasoning presented in the Results section (“Transcriptome-based reconstruction of neurogenesis by striatal astrocytes”).

Cell transplantation experiment (Fig. 5) and EGF injection experiment (Fig. 6):

Sample sizes were based on availability of mice carrying the required genotype.

**Replicates**

- You should report how often each experiment was performed
- You should include a definition of biological versus technical replication
- The data obtained should be provided and sufficient information should be provided to indicate the number of independent biological and/or technical replicates
- If you encountered any outliers, you should describe how these were handled
- Criteria for exclusion/inclusion of data should be clearly stated
- High-throughput sequence data should be uploaded before submission, with a private link for reviewers provided (these are available from both GEO and ArrayExpress)

Please outline where this information can be found within the submission (e.g., sections or figure legends), or explain why this information doesn’t apply to your submission:

Cx30-CreER scRNA-seq dataset:

Cells for this dataset were isolated once, i.e. one mouse for each of the four time points. All 96-well plates with scRNA-seq libraries were prepared side by side and sequenced together, at one single occasion. Cells from the cortex and striatum were isolated from the same mice, as specified in the Results (“Astrocytes outside the striatum initiate an unsuccessful neurogenic program in response to Rbpj deletion”) and Methods (“Library preparation and sequencing for the Cx30-CreER dataset”) sections. Cell inclusion criteria are specified in the Methods (“Read mapping and quality control for striatal cells (Cx30-CreER dataset)”) and in Fig. S1e.

AAV-Cre dataset:

Cells for this dataset were isolated and prepared at a single occasion as specified in the Methods (“Sample preparation for the AAV-Cre dataset”). Thirteen mice were injected with AAV-GFAP-Cre virus. Before mouse brains were processed for library preparation, they were inspected in a fluorescent microscope. At this point, two mice were excluded because they contained recombined cells in the SVZ. Inclusion criteria are specified in the Methods (“Pre-processing of the AAV-Cre dataset”) and in Fig. S2.

Cell transplantation experiment:

This experiment was performed with mice of different strains due to limited availability of mice, and on multiple occasions due to the high workload for each transplantation. Transplantation-recipient mice were excluded from the analysis if they contained <20 transplanted (tdTomato+) cells. The details are specified in the Methods (“Cell transplantation”) and in Table S3.

EGF injection experiment:

This experiment was performed once. A total of 4 mice were injected with EGF in one hemisphere and vehicle in the other hemisphere, as specified in the Methods (“EGF injection”).

**Statistical reporting**

- Statistical analysis methods should be described and justified
- Raw data should be presented in figures whenever informative to do so (typically when N per group is less than 10)
- For each experiment, you should identify the statistical tests used, exact values of N, definitions of center, methods of multiple test correction, and dispersion and precision measures (e.g., mean, median, SD, SEM, confidence intervals; and, for the major substantive results, a measure of effect size (e.g., Pearson's r, Cohen's d)
- Report exact p-values wherever possible alongside the summary statistics and 95% confidence intervals. These should be reported for all key questions and not only when the p-value is less than 0.05.

Please outline where this information can be found within the submission (e.g., sections or figure legends), or explain why this information doesn’t apply to your submission:

This information is given in the main text (see e.g. the section “Stalled neurogenic astrocytes in the striatum can resume neurogenesis if exposed to EGF”).

(For large datasets, or papers with a very large number of statistical tests, you may upload a single table file with tests, Ns, etc., with reference to sections in the manuscript.)

**Group allocation**

- Indicate how samples were allocated into experimental groups (in the case of clinical studies, please specify allocation to treatment method); if randomization was used, please also state if restricted randomization was applied
- Indicate if masking was used during group allocation, data collection and/or data analysis

Please outline where this information can be found within the submission (e.g., sections or figure legends), or explain why this information doesn’t apply to your submission:

Mice were allocated into groups based on genotype (Cx30-CreER dataset, AAV-Cre dataset, cell transplantation, EGF injection), and were age-, sex- and litter-matched when possible (cell transplantation, EGF injection).

**Additional data files (“source data”)**

- We encourage you to upload relevant additional data files, such as numerical data that are represented as a graph in a figure, or as a summary table
- Where provided, these should be in the most useful format, and they can be uploaded as “Source data” files linked to a main figure or table
- Include model definition files including the full list of parameters used
- Include code used for data analysis (e.g., R, MatLab)
- Avoid stating that data files are “available upon request”

Please indicate the figures or tables for which source data files have been provided:

Mouse inclusion data for the cell transplantation experiment is presented in Table S3. All RNA-seq analyses were performed using pre-existing packages as specified in the Methods section.
